# The Application of the Concept of Resilience in Aging Research and Older Adult Care: A Focus Group Study

**DOI:** 10.3389/fmed.2020.00365

**Published:** 2020-08-04

**Authors:** Milou J. Angevaare, A. A. Monnier, K. J. Joling, M. Smalbrugge, F. G. Schellevis, C. M. P. M. Hertogh, M. Huisman

**Affiliations:** ^1^Department of Medicine for Older People, Amsterdam Public Health Research Institute, Amsterdam UMC, Vrije Universiteit Amsterdam, Amsterdam, Netherlands; ^2^Nivel (Netherlands Institute for Health Services Research), Utrecht, Netherlands; ^3^Department of Epidemiology and Biostatistics, Amsterdam Public Health Research Institute, Amsterdam UMC, Vrije Universiteit Amsterdam, Amsterdam, Netherlands; ^4^Department of Sociology, Vrije Universiteit, Amsterdam, Netherlands

**Keywords:** resilience, older adult care, aging, focus group, research approach

## Abstract

**Introduction:** Research incorporating resilience, a concept featuring a positive outcome despite some type of stressor, has the potential to identify possibilities for promotion of the well-being of older people. This study aims to gain insight into the value and potential applications of resilience in both research and care practice from the perspective of researchers and care professionals. Specifically, the value of two scientific approaches, the *a priori* (i.e., based on *a priori* definition of a stressor and outcome) and dynamical systems approaches (i.e., based on mathematically modeled patterns in the real-time response to perturbations), was explored.

**Methods:** Focus groups were performed to explore the thoughts of academic researchers from different disciplines in the fields of aging and care and care professionals on the application of the concept of resilience, including the *a priori* and dynamical systems approaches. Analysis of these focus groups was based on the framework method.

**Results:** Five focus groups were held with a total of nine researchers from different disciplines (e.g., epidemiology, sociology) and 15 older adult care professionals from different professions (e.g., elderly care physician, physiotherapist). The participants described resilience as a concept with value for both aging research and care through its positive connotation and comprehensiveness. Continued research was thought to play an important role in clearing up some of the existing ambiguity surrounding resilience. The importance of resilience in the context of both high- and low-intensity stressors was underscored. The *a priori* and dynamical systems approaches were considered to have their specific advantages and disadvantages on both conceptual and feasibility levels. Therefore, the use of both approaches, side by side and in combination, was suggested.

**Conclusion:** This qualitative exploration among researchers and care professionals confirms that the concept of resilience, including the *a priori* and dynamical systems approaches, is valuable. However, more work is necessary before can be delivered on the potential of resilience in aging research and older adult care practice. Greater conceptual and operational clarity can be achieved through more qualitative studies on the concept that take the perspective of older people into account and through empirical studies that work with both approaches simultaneously and/or in combination.

## Introduction

As a consequence of increasing longevity, there is an increasing interest in promoting the quality of life or well-being in older age (1). Research incorporating the concept of resilience, which refers to situations characterized by a positive or better-than-expected outcome (response) to some form of adversity (stressor), may have the potential to improve older adult care policy and practice (2, 3). The idea behind most resilience research is that there are traits or resources that play a role in achieving the positive outcome (2, 4). It has been suggested that gaining insight into these factors can help to promote quality of life in this population (2, 3).

However, there is a lack of consensus about what resilience is and how it should be investigated in empirical research (2, 5–7). Recent reviews highlight that there are various theoretical approaches to operationalizing resilience (2, 6). The applicability of empirical results in elderly care practice may depend on the chosen approach. Two of the currently most commonly used, distinctive approaches in research in older persons are the *a priori* approach (4) and the dynamical systems approach (8). The *a priori* approach has also been called the definition-driven or researcher-driven approach (2, 6). In this approach, researchers define the two essential components of resilience, the stressor and an outcome (doing better than expected given exposure to that stressor), *a priori* for the specific situation of interest (4, 6). A study subject's resilience is inferred based on this definition. For example, Kok et al. (9) utilized this approach in a study of resilience in older adults with a low social economic status. The stressor was defined as having had a low socioeconomic position throughout life. The positive outcome was an above-average score on a successful aging index, which encompassed trajectories of physical, mental, and social functioning in old age based on cohort data. A number of participants who met these criteria were interviewed about their life. Subsequently, grounded theory analysis was employed to identify themes on how the subjects handled the stressors they encountered throughout their life. The *a priori* approach has also been applied in various other empirical studies in older persons (10–12).

The underlying assumption of the dynamical systems approach is that a person's reactions to daily hassles, perturbations, or stressors (so-called “microrecoveries”) give an impression of a person's overall capacity to recover. These microrecoveries can be captured by monitoring a person in real time (8, 13, 14). Many types of data can theoretically be monitored within this approach as long as the data can realistically be measured repeatedly over time, for instance, with ecologic momentary assessments. The time frame in which the data within this approach is collected varies greatly, depending on the time scale in which meaningful change takes place. This can, for example, be within minutes in the case of a physiological or physical parameter, such as postural balance, to months in the case of a mood or well-being parameter. Determining the correct time frame to measure a specific parameter is essential to the dynamical systems approach (8, 13, 15, 16). This data can subsequently be plotted over time, and specific mathematically modeled patterns are characterized as resilient. Gijzel et al. (8), for example, apply this approach in a study in older long-term-care facility residents using daily self-reported health data measured over 100 days. They observe three data patterns that are indicative of resilience, so-called dynamical indicators of resilience (DIORs): low variance, low temporal autocorrelation, and low cross-correlation. For example, self-reported physical health of someone with high resilience will fluctuate less (low variance) than in someone with a low level of resilience (8). A shorter time to return to equilibrium following disruptions (low temporal autocorrelation) is also indicative of resilience. A resilient person will recover more quickly from a disruption in self-reported physical health. Finally, low cross-correlation entails that, in resilient individuals, disruptions in one system do not necessarily lead to disruptions in other systems. For example, when resilient individuals experience a disruption in their physical health, this does not necessarily lead to a disruption in mental health (8).

The two approaches that we describe here both operationalize resilience but do so in different ways. For this study, we were interested in exploring the opportunities and barriers for application of these two approaches in research and older adult care practice. The aim of this study was to gain insight into the value and potential applications of the concept of resilience in both research and care practice from the perspective of researchers from different academic disciplines and care professionals. We feel that exploring the perspectives of these stakeholders, who typically are not included in discussions on the concept of resilience, may lead to novel insights relevant for older adult care. We primarily explored this for the *a priori* and the dynamical systems approaches to resilience.

## Materials and Methods

### Study Design

A qualitative study involving focus group interviews was performed to explore views and thoughts on the relevance, applicability, and possible applications of the concept of resilience in older persons in general and the *a priori* and dynamical systems approaches in particular, for both research and care practice.

### Participants

We were interested in the applications in science and practice as both are of great importance to improving the care of older people. Therefore, focus groups were held with two groups of stakeholders: (1) academic researchers involved in different disciplines within aging and care research and (2) care professionals within different professions in the care of older people. Purposive sampling among the network of the research group was employed to ensure the recruitment of participants across different fields of expertise, institutions, and professions throughout the Netherlands. Possible participants were sent an email requesting their participation and/or their help in recruiting among colleagues. Experienced older adult care professionals currently employed at a health care organization or hospital in the region of Amsterdam and researchers with different specializations (e.g., sociology, epidemiology, movement sciences, geriatric rehabilitation) within aging and care of older people affiliated to a Dutch university were invited to participate. These researchers were not specialized in resilience and had not worked with the two approaches described here previously. Within the care professional group, we invited both professionals who work with older persons living in the community and those living in long-term-care facilities. Professionals, ranging from district nurses to physiotherapists to medical specialists in the hospital setting, were approached.

### Data Collection

The focus group methodology was chosen because it allows for efficient data collection and provided the opportunity for group discussion allowing participants to build on each other's thoughts and compare experiences. We found this of particular value for this conceptual and relatively abstract topic.

Four initial focus groups of ~90 min each were held, two with researchers and two with older adult care professionals. The focus groups were led by two different moderators with some background knowledge on the approaches (FS for focus groups 1 and 3; MS for focus groups 2 and 4). Prior to each focus group, participants were asked to watch a brief video providing a general introduction on the background of the concept of resilience in science and the two approaches. At the start of each focus group, a researcher (MA) gave an introductory presentation about resilience with a particular focus on the specifics of the two approaches. The content of this presentation was comparable to the description of the approaches in the introduction of this article (see Supplementary Materials for a translated example). MA and MH prepared the introductory video and presentation. MA remained present during the discussions to both observe and assist the moderator.

The topic guide for these focus groups addressed: current recognition or uses of resilience in clinical and research practice, potential advantages and disadvantages, and possible research and older adult care applications of resilience. Both the *a priori* and dynamical systems approaches of resilience were explored. The topic guide and introduction were refined after each focus group, following the principles of research as an iterative and reflexive process (17).

Finally, a fifth focus group of ~60 min was held with a combination of participants of the previous focus groups consisting of both researchers and care professionals, moderated by FS. Again a variation in profession and area of expertise was sought. During this focus group, preliminary results from the first four focus groups were presented to the participants, and the participants were asked to reflect on and react to these results. Thus, this final focus group functioned as respondent validation or a “member check” for the preliminary results (17).

The focus groups were conducted in Amsterdam and Utrecht, the Netherlands, from January until March 2019. Subsequently, audio recordings of each focus group session were transcribed verbatim.

### Analysis

The transcripts were anonymized before being analyzed in the computer-assisted qualitative data analysis software ATLAS.ti. Analysis was based on the framework method for the analysis of qualitative data in multidisciplinary health research (18). An “*in vivo*” coding strategy (17) was applied to reflect the terminology used by the participants. Two focus groups were independently coded by two researchers trained in qualitative research (MA, AM). The resulting codes were discussed and organized during face-to-face research meetings until agreement on a working analytical framework was reached. This working analytical framework was subsequently applied to the coding of the other three focus groups by one researcher (MA). Addition of codes and changes to the analytical framework were made in agreement with a second researcher (AM). The interpretation of the data and patterns emerging from the data were discussed within a group of four researchers (MA, AM, CH, and MH).

A combined thematic analysis approach was undertaken: Themes were both inductively established from participant accounts and were guided by our main research questions: What are the views and thoughts of experts on (the applicability of) resilience in older persons? What are possible advantages and disadvantages of the *a priori* and dynamical systems approaches? What are possible applications of resilience and the two approaches in research and practice? The complete analytical framework including all codes is available upon request.

### Ethical Review

Each participant gave written informed consent prior to participating in the focus groups. The medical ethics review committee of VU University Medical Center assessed the study protocol and concluded that, according to Dutch legislation, it was exempt from their approval; reference number 2018.527.

This report was composed in accordance with the consolidated criteria for reporting qualitative research (COREQ) (19).

## Results

Twenty-four participants, nine researchers, and 15 care professionals participated in the first four focus groups. Table 1 provides an overview of the research disciplines and older adult care professions represented by the focus group participants. The six participants of the final member check focus group agreed with the preliminary results as presented in this focus group and were able to elaborate on the preliminary themes. As the input from the researchers and care professionals showed a high level of agreement, their input was analyzed collectively.

**Table 1:** Research disciplines and professions represented by the focus group participants in no particular order.

**Focus group 1: care professionals**	**Focus group 2: researchers**	**Focus group 3: researchers**	**Focus group 4: care professionals**	**Focus group 5: care professionals & researchers**
2 clinical psychologists	2 care of older people & welfare	1 epidemiology	4 elderly care physicians	1 clinical psychologist
1 district nurse	1 care of older people	1 geriatric rehabilitation	1 clinical psychologist	1 elderly care physicians
1 elderly care physician	1 (medical) humanities	1 (medical) humanities	1 internist-geriatric medicine	1 occupational therapist
1 nurse practitioner		1 movement sciences	1 occupational therapist	1 researcher epidemiology
1 physiotherapist		1 sociology	1 physiotherapist	1 researcher geriatric rehabilitation
1 senior advisor				1 researcher (medical) humanities

Three main themes were identified in the analysis: the concept of resilience in older persons, the *a priori* and dynamical systems approaches, and the application of resilience in research and older adult care practice. Within each theme, several subthemes were identified (Table 2), which are presented below and are substantiated by verbatim quotes.

**Table 2:** Overview of identified themes and subthemes.

**The concept of resilience in older persons**
Interpretation of resilience
Added value of resilience
Finiteness of resilience
**The *a priori* and dynamical systems approaches**
Recognition/comprehensibility/intuitiveness
Stressor
Judgment
Feasibility
Use of both the *a priori* and the dynamical systems approaches
**Application of resilience in research and older adult care practice**
Application of resilience in research
Application of resilience in care practice

### The Concept of Resilience in Older Persons

#### Interpretation of Resilience

There were many different interpretations of what resilience entails and its most important components or contributing factors.

Recovery, resistance, acceptance, anticipation, compensation, self-management, and reflection were all described by the participants to play a role in resilience. Resistance and recovery were mentioned as aspects of resilience in the introductory presentation. Resistance to the occurrence of (the negative effects of) a stressor and recovery from those effects were subsequently acknowledged by participants to be essential to resilience (research). Essential to the recovery aspect of resilience is the level of recovery that is expected in the context of resilience. Participants felt that different stable states, e.g., levels of functioning, can be achieved in the recovery process. It is not always necessary or even advantageous to remain at or return to the same state as prior to the stressor. Participants described that a new state can be indicative of resilience as long as it remains stable over time. Acceptance can play an important role in this type of resilience: being successful in a different state, e.g., a lower level of functioning, than before. In short, dealing with stressors requires adaptability, and this adaptability was described to be an important aspect of resilience.

“Of course it is also possible that you stay at that level, and that you remain stable and simply continue. That's fine of course. You don't have to come back to exactly where you were because that is not always the most ideal situation.” (researcher 9, fg3)

In discussions on the resistance aspect of resilience, anticipation was described to play an important role. Indeed, it was suggested that the exposure to a stressor or the negative effects of this exposure could be avoided by thinking and acting proactively. Participants also believed that anticipation was closely related to compensation for losses, such as loss of independence, at this age. This anticipation was described to encompass two aspects: reflection and action/organization. Different specific examples of anticipation in the context of aging were given, including building a social support system, proactively making changes in the home or moving to a single-story house, training different skills, and remaining interested in the environment.

“Yes, and there, this implies in a practical sense that you organize it, but also that you are already preparing in your mind for what […] That things may get worse and how that would be for you, and yes, of course this can make you all depressed, but it can also help you cope with it later on, but well, how do you measure that? And there may be many more aspects to this anticipation. Of course there are people who will, uh, start training really intensely to prevent them from, uh, deteriorating, in all areas.” (researcher 6, fg5)

Personality and coping style were described as important sources of resilience. For many participants, social support, the social network, and especially reciprocal relationships, were additionally highly important to resilience on a contextual level.

“I may have a really good example of that. Of someone who, uh…, I teach fall prevention classes these days. […] And this lady, she was always at home by herself, and no social contacts either. And she joined this group, and it was a very nice and warm group. And she says, now I have twelve new friends. She applies for all kinds of classes at once, and she goes from a to b. And all the people who didn't treat her well, because they walked all over her, she ditched them. And she sort of created a whole new life, which increased her quality of life.” (care professional 6, fg1)

Examples of other contributing factors mentioned during the focus groups were previous experience with dealing with stressors, level of physical activity, and level of education.

#### Added Value of Resilience

Although resilience was repeatedly described by the participants to be similar to other related concepts, such as coping, frailty/physical reserve capacity and self-management, most agreed that resilience is of added value compared to these other concepts through its comprehensiveness and positive connotation. Resilience was described as more comprehensive than other constructs by not only including individual characteristics, such as personality and coping strategies, but also incorporating characteristics related to time and context.

“[…] I see resilience much more as sort of a systems approach: what it is that makes… and then you do justice to the situation, and to that social network, and that context, and that moment in time. And that, I feel, makes it more complete than, uh, those coping strategies.” (researcher 4, fg 2)

In contrast to concepts such as frailty, resilience was reported to have a positive connotation as it focuses on recovery/the opportunity for a positive outcome in light of stressors and not on the negativity of the stressor itself. It also incorporates the potential of improvement through, e.g., interventions. Finally, resilience was described to have a holistic nature, implying more than just overcoming a single challenge.

“But the resilience model is of course nice and abstract, just like positive health, but that it allows you to, uh, create a thinking model in which it makes sense that you go a little further than just treating the disease. That you also help someone toward a positive health to increase his resilience so he doesn't get sick again as quickly and you have to do all kinds of things again.” (care professional 11, fg5)

#### Finiteness of Resilience

Another recurring theme within the discussions of the concept of resilience was its finiteness. The idea that resilience has limits was brought up in all focus groups; however, different interpretations of this finiteness were given. It was described in relation to increasing age, imminent death, and a high number or intensity of stressors. Loss of meaningfulness, self-management, physical condition, and motivation were all thought to play a role in this finiteness. Older persons were described as being able to sense this end of resilience. Participants believed that, in some cases, it reflected a type of withdrawal from life.

“More like what you say, flexibility diminishes, which literally means you lose your resilience. An older person, yes in the beginning, I imagine, the line goes like this [indicating a stable line], and at some point it starts to fluctuate a lot more because this flexibility is gone.” (care professional 15, fg4)

Participants also discussed whether resilience really ends or if resilience may actually take on a different form in some situations. This tied in with the expected level of recovery as described above: One does not always have to remain in the same state, e.g., the same level of functioning, to be resilient. For example, actively coping with challenges may turn into acceptance of one's decline and end of life, and this may be indicative of resilience as well. Thus, some participants feel that, in this situation, resilience does not end, as it were, but takes a different form.

“Of course, there are other ways to go forward then. It doesn't have to be like it was every time before, uh, that you think, oh I will get back to a certain level, uh, but then move forward again on a different level.” (care professional 5, fg5)

[….] “Yes, that has more to do with the aspect of giving meaning to life, that a person, even in a new role in which his ability or willingness to do things independently is decreased […], can still find happiness.” (care professional 11, fg5)

### The *a priori* and Dynamical Systems Approaches

Table 3 provides an overview of advantages and disadvantages of the *a priori* and dynamical systems approaches as described by the participants. Below, we give a more extensive description of the participants' considerations regarding these two approaches.

**Table 3:** Overview of advantages and disadvantages of the *a priori* and dynamical systems approach as seen by researchers and care professionals.

	**Advantages**	**Disadvantages**
*a priori* approach	•Recognizable, easy to understand and translate to intervention •Feasible, possible with existing data	•Less flexible, stressor pre-determined •More judgmental •Only incorporates reaction to one stressor •Emphasizes limitations •Too broad, anything can be encorporated
Dynamical systems approach	•Studies disturbances in daily life •Flexibility in timescales •Incorporates resistance and recovery •Patterns feel less judgmental •Innovative •Nuanced •Organic •Includes resignation	•Difficult to understand /abstract •Circumstances & stressor unknown •Intensive data collection

#### Recognition/Comprehensibility/Intuitiveness

The *a priori* approach was seen to be intuitive and easy to understand. Furthermore, especially care professional participants saw a clearer translation to practice as compared to the dynamical systems approach because there is a strong connotation of possibilities for intervention. At first glance, the dynamical systems approach was more difficult to understand; it was considered to be more abstract compared to the *a priori* approach.

Additionally, some participants described that the resilient patterns within the complex dynamical systems approach do not correspond with their own interpretation of resilience. Specifically, the “low-variance” pattern was counterintuitive. A “dip” after a more intense stressor, which would imply high variance in the data, was actually seen as healthy by most participants. Therefore, rapid recovery from such a dip, characterized by low temporal autocorrelation in the data, was felt to be more in line with resilience than not having these “dips” altogether.

“That you can actually say that the degree of recovery […] say, the time it takes for you to climb back up, actually says more about your resilience than remaining stable on a straight line.” [in response to resilient patterns in the complex dynamical systems approach] (researcher 7, fg3)

The low-variance pattern also did not reflect thoughts on a healthy balance between variability and stability: complete stability and too much variability both were seen as disadvantageous to resilience by participants. In other words, a certain amount of baseline variance is important for many types of parameters. For example, in the case of balance parameters, complete stiffness is undesirable; a healthy person constantly shifts to determine their position in space.

“What we also see, for example with balance, is that if you indeed lack variation or are too stiff, uh, you know. We always think that limited variation is good, but you also need to be able to explore your limits and have some variability to also be able to resist. so I think that an overly rigid line is not good either.” (researcher 9, fg3)

#### Stressor

Stressors were seen to be an important part of the concept of resilience. According to the participants, consideration of stressors is what differentiates the concept of resilience from functioning in general. Two different types of stressors were recognized: a high- and a low-intensity stressor. A high-intensity stressor may include loss (e.g., of a partner), disease, amputation, or past trauma. It is in the nature of *a priori* approaches that this type of stressor is clearly defined as the context in which resilience takes place. This may be part of the reason that this approach was more recognizable for many participants. However, the definition of the stressor was also seen as a disadvantage; it makes this approach less flexible.

“Then I recognize what you are saying. The *a priori* [approach] is of course a bit, it is defined a little more sharply and is therefore less flexible, yes and resilience is basically about flexibility, I think.” (researcher 2, fg2)

As described above, both resistance (to the occurrence of and/or negative effects of a stressor) and recovery were seen as important aspects of resilience. Participants agreed that the recovery aspect of resilience can be studied using both approaches. However, resistance to the occurrence of a stressor can only be studied with the dynamical systems approach, precisely because the stressor is not defined. Thus, resistance to stressors is incorporated in the low-variance pattern of resilience. In other words, avoidance of stressors leads to a more stable pattern. This opportunity to incorporate resistance was described as an advantage of the dynamical systems approach.

“What I see here in the dynamical systems approach, more than in the *a priori* one I think, is that it comprises two factors. Namely what you had in your definition, ability to resist and recover. Uh, this also is. Here you also see when there is no adversity, you don't measure that. But that is the ability to resist that that this adversity affects you or occurs at all. And then I am thinking from the uh, physical, uh, perspective, for example balance. […] If I disrupt a person's balance, then we very often look at how can someone recover. But the question how well can a person actually withstand such a disruption of balance. That is far more important. But that is not what you measure, because that has not taken place.” (researcher 9, fg3)

Throughout the day, there are constantly small natural perturbations that require an individual to adapt. According to several participants, resilience is not only portrayed in response to high-intensity stressors, but also in the reaction to these low-intensity stressors or disturbances in daily life. Therefore, these participants felt that an advantage of the dynamical systems approach is that it studies these disturbances in daily life.

“What I like about it is uh, what is actually also my starting point in my own research. Is that uh, uh, I think it is good to start not only from adversity and then how people adapt. But that there is actually always something to adapt to. That is a little more compatible with, with the dynamical systems approach.” (researcher 8, fg3)

#### Judgment

In the *a priori* approach, the desired outcome after exposure to a stressor is defined *a priori*, and in the dynamical systems approach, the resilient patterns are described as a mathematical characteristic of a system. As a result of this, the dynamical systems approach felt less judgmental to participants than the *a priori* approach.

“I really, I really like to be able to explain how things go the way they go. And that you can say that there are patterns people can fit into. That that can also be reassuring to people. So if you can explain they are not different from others, which is often how they feel.” (care professional 6, fg1)

#### Feasibility for Research

The *a priori* approach can be applied retrospectively with existing (cohort) data, making it a relatively feasible approach for research. The dynamical systems approach, on the other hand, requires specific collection of data at many different time points, which was seen as more cumbersome by participants.

#### Use of Both the *a priori* and Dynamical Systems Approaches

In short, both approaches were recognized to have their own value by the participants. As a consequence, an interest in using both approaches to study resilience was expressed in all focus groups. These two approaches may be employed in different situations. For example: many participants felt both types (high and low intensity) of stressors to be important, but the resilience that is portrayed in reaction to these stressors may be different. In other words, resilience in the context of these two different stressors may represent different types of resilience. Therefore, the different approaches may be used to study resilience in reaction to different types of stressors.

“Because for different types you also need different approaches, different solutions, etc. And I think the same will prove to be true for resilience.” (researcher 1, fg2)

According to participants, another way to utilize both approaches is by combining (aspects of) them. For instance, by applying the dynamical systems approach around the time of a high-intensity stressor or measuring them both within one individual at the same time.

### Application of Resilience in Research and Older Adult Care Practice

#### Application of Resilience in Research

Participants described different considerations for the application of resilience in research.

The fact that there is no clear definition of resilience was reported to complicate research. Future research on the concept of resilience may help resolve some of this ambiguity.

“Yes, and then especially, that is the beauty of it, that it is being studied to make sure, or to prevent that it becomes one of those catch-all terms, but rather to put more flesh on it.” (researcher 3, fg 2)

Furthermore, it was put forward that resilience may be so person-specific that it may be impossible to generalize resilience to populations.

“Or it varies so much from person to person that in the end you can't really say anything about it. That is also a possibility, I think, but well.” (researcher 7, fg5)

A preference for longitudinal data collected specifically for resilience research as opposed to the use of existing data was expressed.

A recommendation was to do justice to the subjective nature of resilience in resilience research. Three different aspects of this subjectivity in resilience (research) were described. First, the perspective of the subjects themselves is important for both the discussion of what a stressor is and what a resilient response is in the context of resilience. Second, on an individual level, the meaning that someone attaches to certain aspects or activities in their life are of importance to the role of these aspects in achieving resilience. Last, it was suggested that the quest to clarify the use of the approaches in studies would benefit from input of (representatives of) the subjects themselves.

“But then I would also want to know, what are people themselves saying about it. Actually that question we have [discussed] here before. What are people saying about how they function, about how they deal with adversity.” (researcher 7, fg3)

The participants were specifically asked to think about data that can be used within the dynamical systems approach. Self-rated health and balance measures and measurement using wearables were given as examples in the introduction of the focus groups. The participants themselves suggested quality-of-life data, gait pattern, well-being data (experience sampling), registration data in general practice, mood measurement, positive health data, sleep patterns, and voice patterns (volume, speed, and/ or intonation).

Participants described several considerations concerning the application of the dynamical systems approach. There is a necessity of repeated measurement data. Therefore, the data should be relatively easy and quick to collect with little burden to the participant. Repeated measurements may lead to learning effects. Cognitive data, for example, may, therefore, be difficult to collect. Implicit measurement can help to prevent this and other types of bias. Ambulant monitoring of behavior (with wearables) was recognized as a good way to apply implicit measurement.

Tying in with the holistic character of resilience, multifactorial data should be considered within the dynamical systems approach. Although, this applies for all resilience research, it was stressed for the dynamical systems approach in particular. This importance is also implied by the inclusion of the resilience pattern of low cross-correlation (low correlation between patterns across different systems, e.g., mental and physical health) within this approach. Determining resilience using one data type may give a distorted view. Other functional systems that are not captured by the data may either compensate for or disrupt the resilience portrayed by this one data type. In one focus group, the different domains within the positive health concept described by Huber et al. (20) were seen as ideally suited to function as a guideline for this combination of data.

#### Application of Resilience in Care Practice

Aspects of resilience and, specifically, the description of resilience within the *a priori* approach were recognized in care practice, particularly within the geriatric rehabilitation setting. In this setting, everyone is recovering from some sort of high-intensity stressor.

Intervention and clinical decision making were seen as two important applications of resilience in practice. Knowledge of the factors that play a role in resilience can be used to map someone's strengths and weaknesses. This can, in turn, give direction to intervention or justification for clinical decision making.

“Well, what I think we were just saying, that you, this, that this is part of what you could include in, uh, diagnostics and drawing up a treatment plan. So mapping it out. So I think that is where it starts, and the question is whether this is always something we can influence, but it starts with mapping.” (care professional 14, fg4)

Thus, there is a keen interest in factors that play a role in resilience. With knowledge of these factors, care professionals can influence them to improve resilience. Relative weaknesses can be improved on by specific interventions. For example, social support can be improved by advising participation in social activities. On the other hand, strengths, such as optimism, can be called upon by care professionals in the recovery process. This type of influencing of resilience is currently being applied in daily practice by, for example, psychologists, physiotherapists, and occupational therapists but often quite implicitly. This could be done more explicitly; for example, resilience assessment can play a specific role in the development of personal treatment plans in accordance with the principles of personalized medicine.

“Not very consciously, I think, but I think sometimes with people you do … if you think about it a little, then you know like, these are resilient [persons] in my opinion. And when I think of the *a priori*, I can also see why that is. So whether [this person] is optimistic, or [they] have always been very active physically, for example. If you think about it like that, then they are there. But I don't think we are always aware of it.” (care professional 9, fg4)

“My background is of course one of medical training. So then the logical places to start are, uh, start with exercise, start with nutrition. These are measures of resilience I think I can influence. Increasing your social network is also something we recommend, uh, activities, day center. Uh, uh, that kind of thing, yes.” (care professional 10, fg4)

Besides using resilience to give direction to the use of existing interventions, resilience research may also lead to the development of specific interventions to improve resilience.

At the basis of resilience interventions is the discussion on if resilience can be influenced. Most participants agree that resilience can be influenced both by individuals themselves and by interventions or treatment. This potential to influence resilience may be limited by different factors, such as age and total number of stressors. However, as described earlier, resilience is also seen as something finite by some. Thus, at a certain point resilience ends and with it the possibility to influence it.

Knowledge of a person's resilience can also inform clinical decisions that are not necessarily meant to improve resilience. Assessing someone's resilience can help predict a subject's course over time, thereby informing clinical decision making, for example, in the context of starting cancer treatment or performing an operation and triage for rehabilitation.

“I immediately start thinking about treatment goals. […] We are asked frequently in the policlinic and the clinic whether we think an individual is still able to undergo an, uh, aortic valve replacement, or to get chemotherapy or not, or to undergo surgery. To think about how far you take medical treatments. Well, in that case you naturally do a full examination of a person. But, I also think that you consciously try to determine: is this person able to handle this, and perhaps subconsciously that means: is a person resilient enough.” (care professional 10, fg4).

## Discussion

Academic researchers and care professionals believe the concept of resilience to be valuable for aging research and older adult care and are positive about both the *a priori* approach and dynamical systems approach. Resilience is seen as being similar to other concepts, such as frailty. However, its specific value is believed to lie in its positive connotation through the implication of the possibility for recovery. Furthermore, participants felt that the concept does justice to the whole person and incorporates time and context. However, there is still a lot of ambiguity surrounding resilience. During the focus group discussions, this was evident from the large amount of different associations resilience evoked among the participants. Examples of different associations that were evoked were adaptation to new states and resistance to stressors through anticipation and acceptance. In contrast with the interest in anticipation in some focus groups, the incorporation of a stressor was also seen as essential and distinguishing to the concept of resilience. The participants further described that continued research, such as the current study, can help clear up some of the existing ambiguity. The importance of resilience in the context of both high- and low-intensity stressors was underscored. The *a priori* and the dynamical systems approaches were described to each have their specific advantages and disadvantages on a conceptual and a feasibility level. The application of these two approaches was expected to yield different but valuable types of information.

Contrary to expectations, no real differences or contrasts were found between the viewpoints of the researchers and care professionals. Both groups of stakeholders had very comparable ideas about the application of resilience and the two approaches; therefore, their input was analyzed collectively.

Participants described the dynamical systems approach to feel less judgmental than the *a priori* approach. However, although this was not discussed during the focus groups, it can be argued that the dynamical systems approach also incorporates a norm through the patterns that are considered to be resilient. In other words, the normative aspect may be hidden within the mathematical model.

### Comparison With Existing Literature

In a reflection of the scientific literature, the concept of resilience itself and its aspects were the subject of much discussion during the focus groups.

Many of the aspects of resilience highlighted by the participants during the focus groups were similar to discussions surrounding resilience in the scientific literature. For example, the importance of the social and broader context of a person, and especially the level of social support they experience, to their resilience is often emphasized in both empirical and conceptual literature on resilience in older persons (5).

The participants pointed to the need to involve older persons themselves in the discussion of what resilience is and how it should be applied or investigated. The importance of the inclusion of this stakeholder group when conceptualizing resilience was also described by literature reviews (5). Indeed, a Delphi study of features of resilience of informal caregivers of persons with dementia demonstrates that the perspective of caregivers themselves on the features of resilience differ considerably from that of professional experts (21).

Aspects of resilience are already routinely applied in the care of older people. Often this is done implicitly, but participants saw opportunities to work more explicitly with resilience. The most important opportunities for the contribution of resilience as described by the participants were improvement of resilience through intervention, clinical decision making, and triage for rehabilitation. A recent review of the dynamical systems approach and clinical practice argues for a more explicit exploration of resilience in geriatric medicine. Although resilience is often implicitly assessed as part of the clinical management of older persons, the review suggests that this clinical management can be improved by the explicit use of the dynamical systems approach of resilience (14).

The participants touched upon the related concept of “positive health” as described by Huber et al. (20). Positive health is conceptualized as “the ability to adapt and to self-manage in the face of social, physical and emotional challenges.” The six dimensions within the concept of positive health, namely bodily functions, mental functions and perception, spiritual/existential dimension, quality of life, social and societal participation, and daily functioning, are suggested to also be useful in guiding multifactorial resilience research.

Additionally, the participants suggested combinations of the two approaches. A recent article portrays a first example of a combination of the approaches as suggested by the participants during the focus groups (22). This study applied both the *a priori* and the dynamical systems approach at the same time surrounding a high-impact stressor.

The interest in and thoughts of the participants on the finiteness of resilience and the importance of anticipation in resilience in older adults have not been extensively described in the literature. Participants associated resilience with anticipation of stressors through reflection and taking measures to prevent them or their effects. In general, within the scientific literature, a stressor is seen as essential to the portrayal of resilience (3). Although avoidance of a stressor has been mentioned to be an aspect of resilience (23), others specifically argue against this (24, 25).

### Strengths and Limitations

This study has several strengths. It is, to our knowledge, the first qualitative study on the applicability and application of different approaches of resilience. This study provides an initial glimpse into what the value of resilience and the *a priori* and dynamical systems approaches in particular can be to aging research and older adult care practice according to experts working in these fields. Also, the study includes the input of participants from many different backgrounds. Experienced academic researchers in different disciplines within aging and care and experienced older adult care professionals from different professions took part in the focus groups. This allowed for a broad and in-depth exploration of the implications of resilience for both research and care practice.

This study also presents some limitations. First, by design, it fails to present a comprehensive inventory of all potential applications of resilience in both research and care practice for older people. Instead, the qualitative exploration yields several illustrative examples of (potential) applications and allows experts to reflect on them and the added value for care practice. Second, participants made several associations between resilience and related concepts (e.g., frailty, coping) and extensively discussed how these related to each other. Subsequently, in the coding process, it appeared difficult to make a clear-cut distinction between interpretations, similar concepts, factors, aspects, components, responses, and outcomes of resilience. As a result, an *in vivo* coding strategy was applied to stay as close as possible to the phrasing used by the participants. Third, the introduction presentation, although prepared with attention to objectivity, might have influenced the discussions in the focus groups (e.g., terminology used by participants).

### Conclusion and Future Research

The participants of the focus groups described both the concept of resilience and specifically the *a priori* and dynamical systems approaches to be of value for aging research and older adult care practice. However, the current ambiguity surrounding the concept and application was both recognized by the participants and evidenced by the large amount of different associations that the participants had with resilience. Therefore, much work is to be done before it can be delivered on the full potential of resilience in aging research and older adult care settings. Greater conceptual and operational clarity can be achieved through more qualitative studies, such as the current study; older persons themselves, in particular, should be included in the discussion. The specific value of the approaches can be explored further through empirical studies that work with both approaches side by side and combine them in different ways.

## Data Availability Statement

Because of the nature of the data (focus group transcripts) these cannot be fully anonymized. The complete codebook including all codes will be available upon request.

## Author Contributions

MA, KJ, CH, and MH developed the project. MA and MH developed the topic list and introductory presentation and video for the focus groups. MS and FS facilitated the focus groups as a moderator. MA and AM performed initial coding, data analysis, and drafted the manuscript. All authors contributed to the finalization of the manuscript.

## Conflict of Interest

The authors declare that the research was conducted in the absence of any commercial or financial relationships that could be construed as a potential conflict of interest.
