# Supplementary material for: The Application of the Concept of Resilience in Aging Research and Older Adult Care: A Focus Group Study
Source: Front Med (Lausanne). 2020 Aug 4;7:365. doi: 10.3389/fmed.2020.00365 (PMC7417312; doi:10.3389/fmed.2020.00365)
Supplement: Supplementary file 1 [file Presentation_1.pdf]

Amsterdam UMC
Universitair Medische Centra

Resilience among older adults:
The value of different resilience approaches

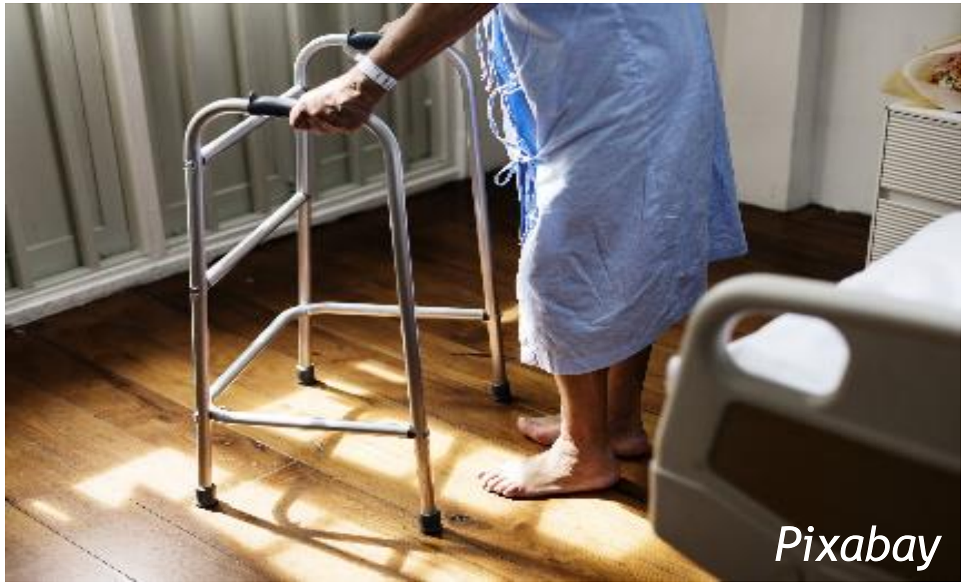

Contents

- Introduction resilience
- Dynamical systems approach
  - Examples
- A-priori approach
  - Examples
- Summary

Resilience: doing well despite adversity

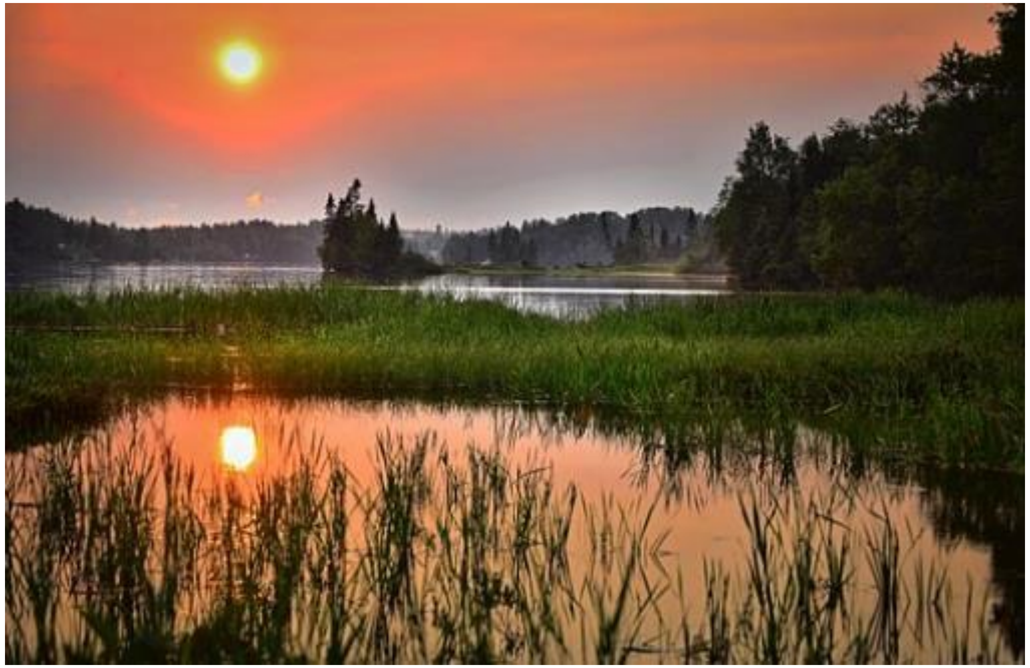

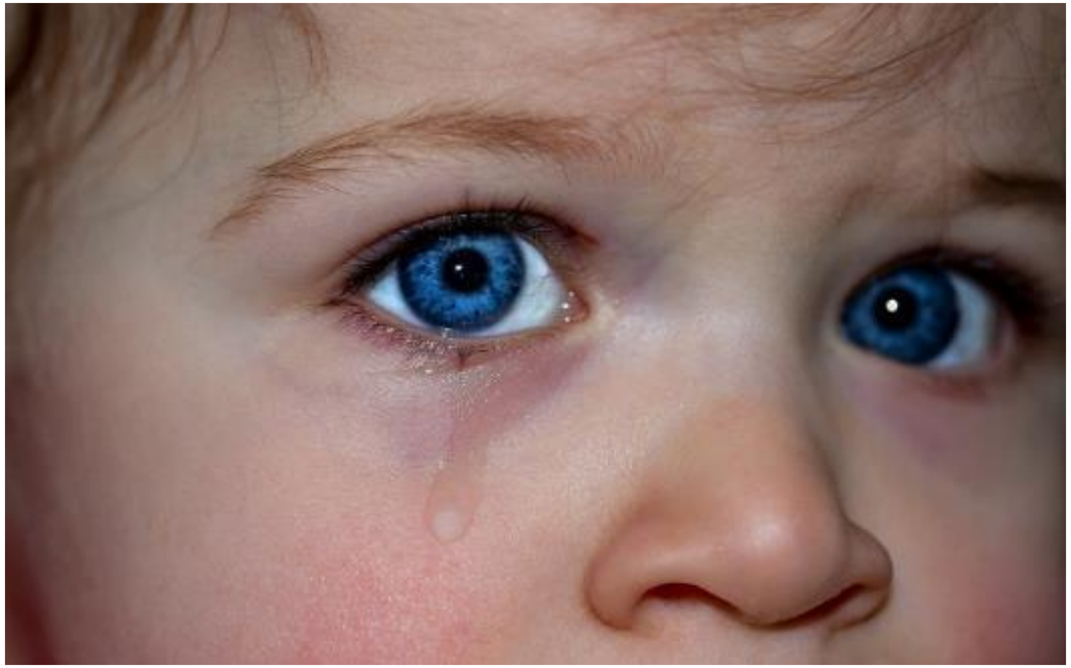

Pixabay

Dynamical systems approach

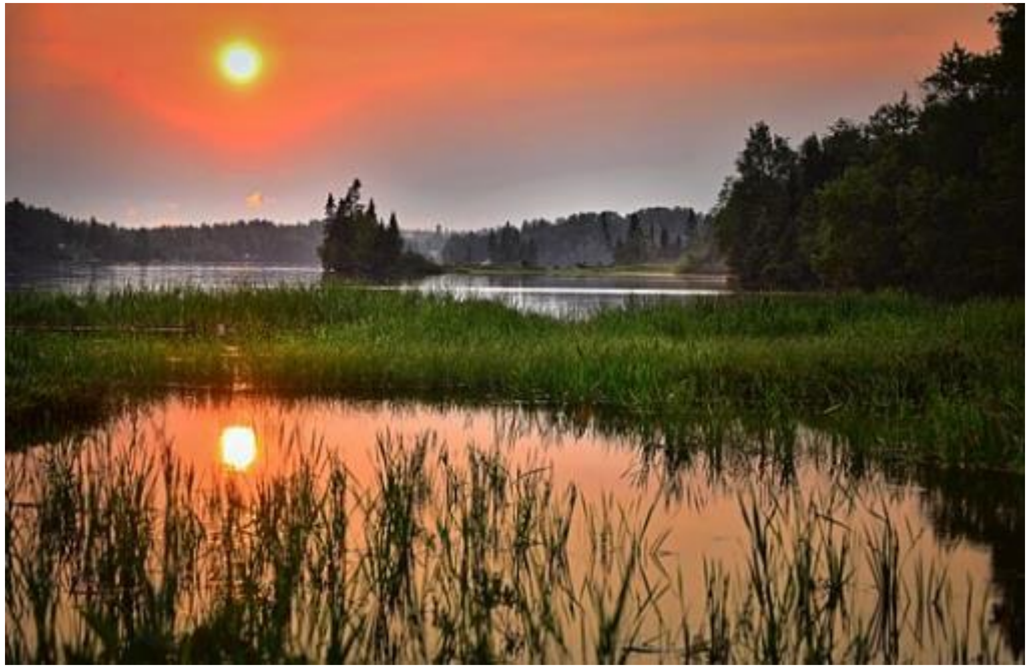

Pixabay

Dynamical systems approach

"An individual's ability to resist functional decline or recover physical health following a stressor."

Gijzel et al. 2018
Whitson et al. 2016

Dynamical systems approach: complex systems

Monitoring system → Reactions to perturbations → Data time series → Patterns

Resilience

Universal Patterns = Dynamical Indicators of Resilience (DIORs)

Validation

Gijzel et al. 2018

Examples

Gijzel et al. 2017

Physical

Mental

Social

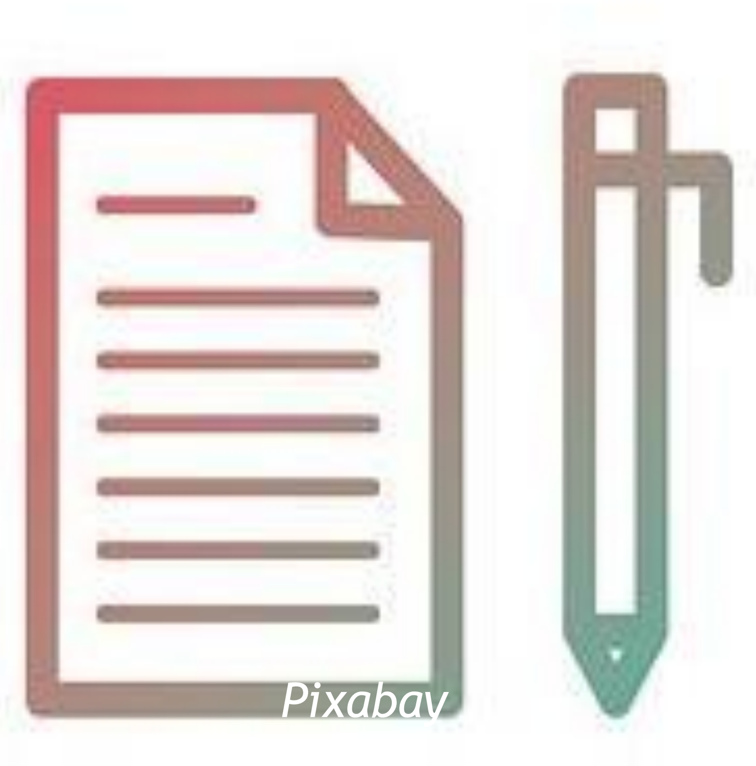

100x: Health?

Dynamical Indicators of Resilience (DIORs)
Universal patterns

Low variance **Low temporal autocorrelation** **Low cross correlation**

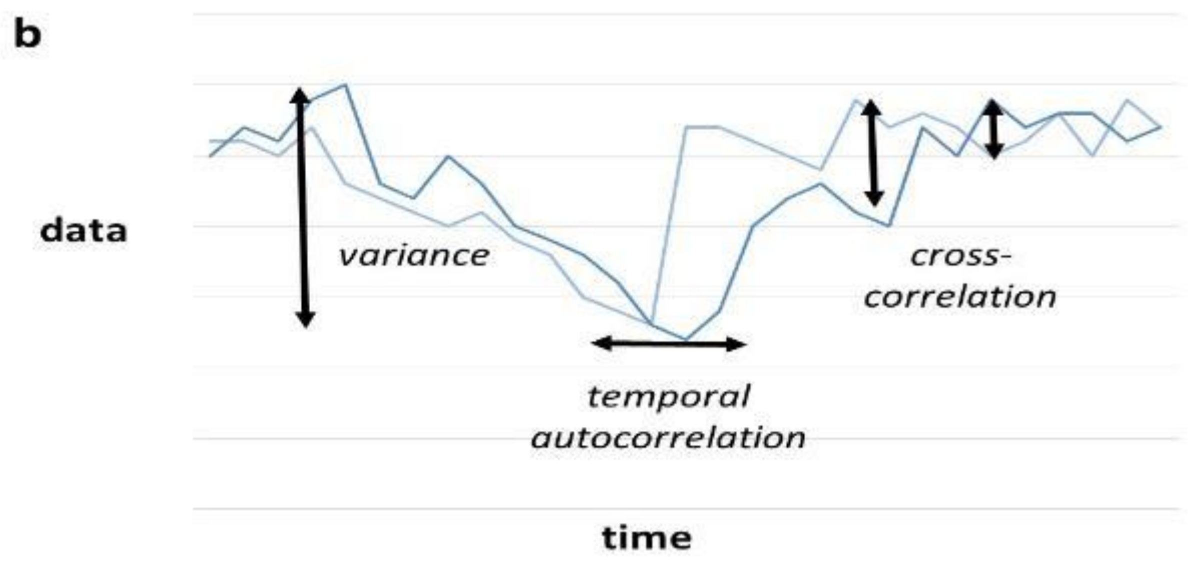

See original graph in Gijzel et al. 2017

Example

Gijzel et al. 2018

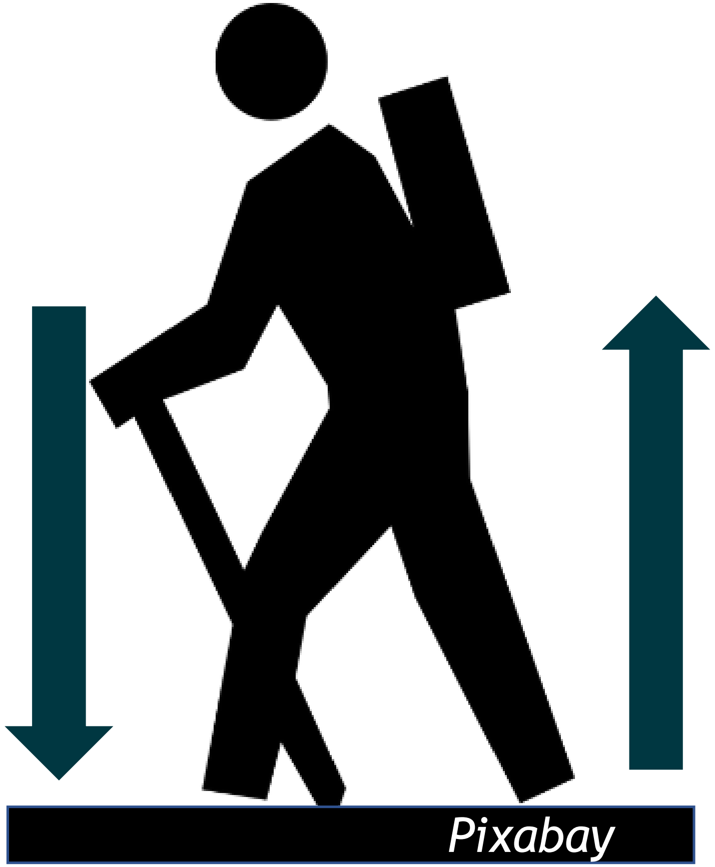

Future applications:
-wearables
-ecological momentary assessment

A-priori approach

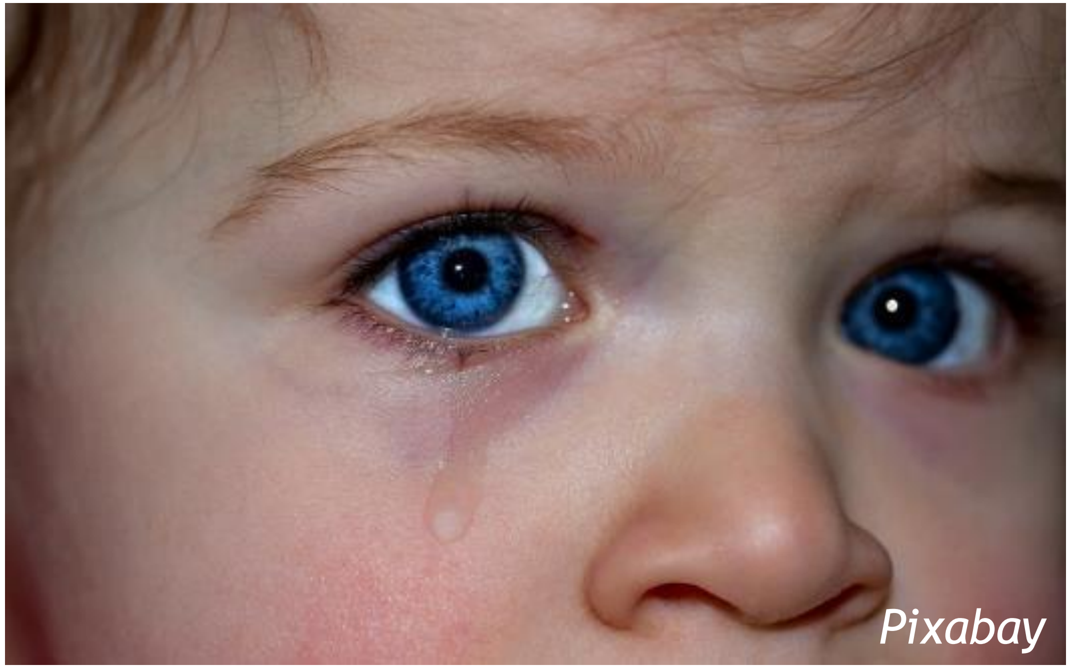

A-priori approach

“Resilience is defined as the process of effectively negotiating, adapting to, or managing significant sources of stress or trauma. Assets and resources within the individual, their life and environment facilitate this capacity for adaptation and ‘bouncing back’ in the face of adversity. Across the life course, the experience of resilience will vary”

Windle 2011

A-priori approach

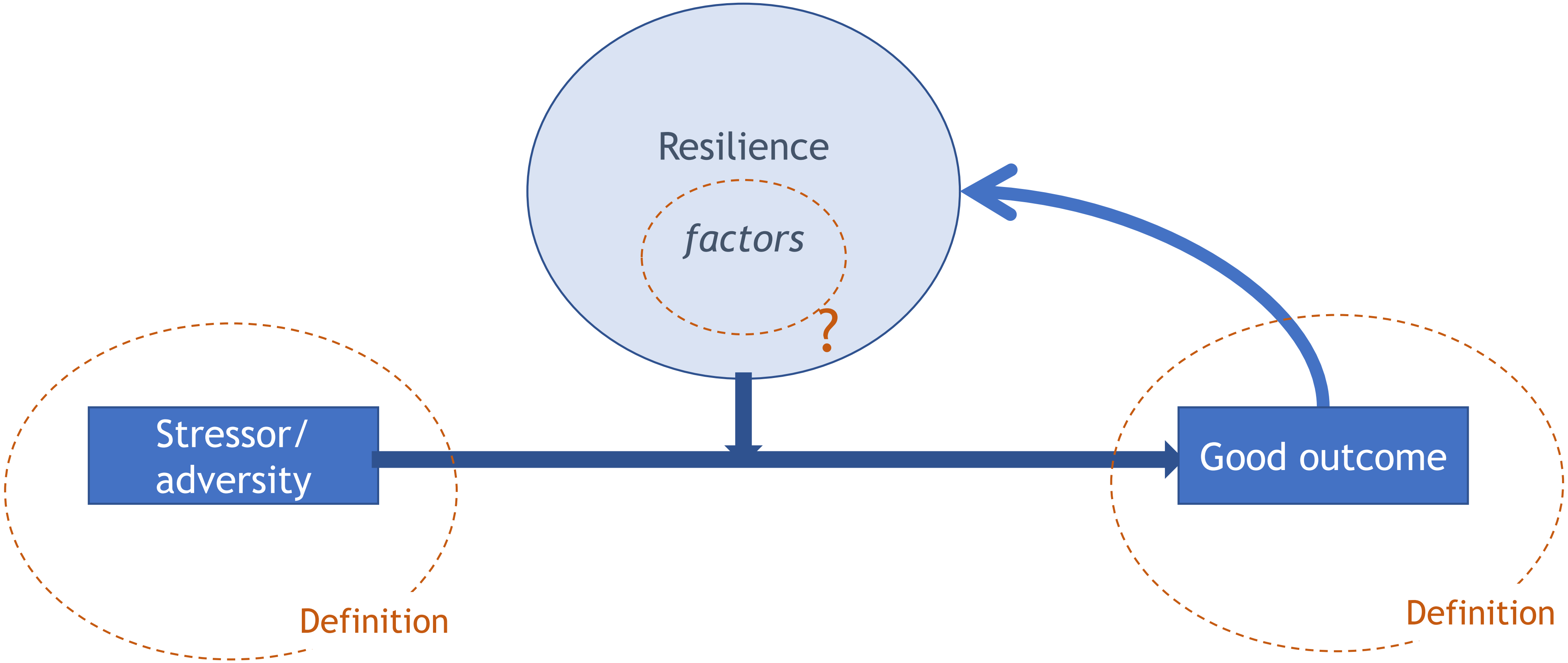

Example: quantitative *Hildon et al. 2009*

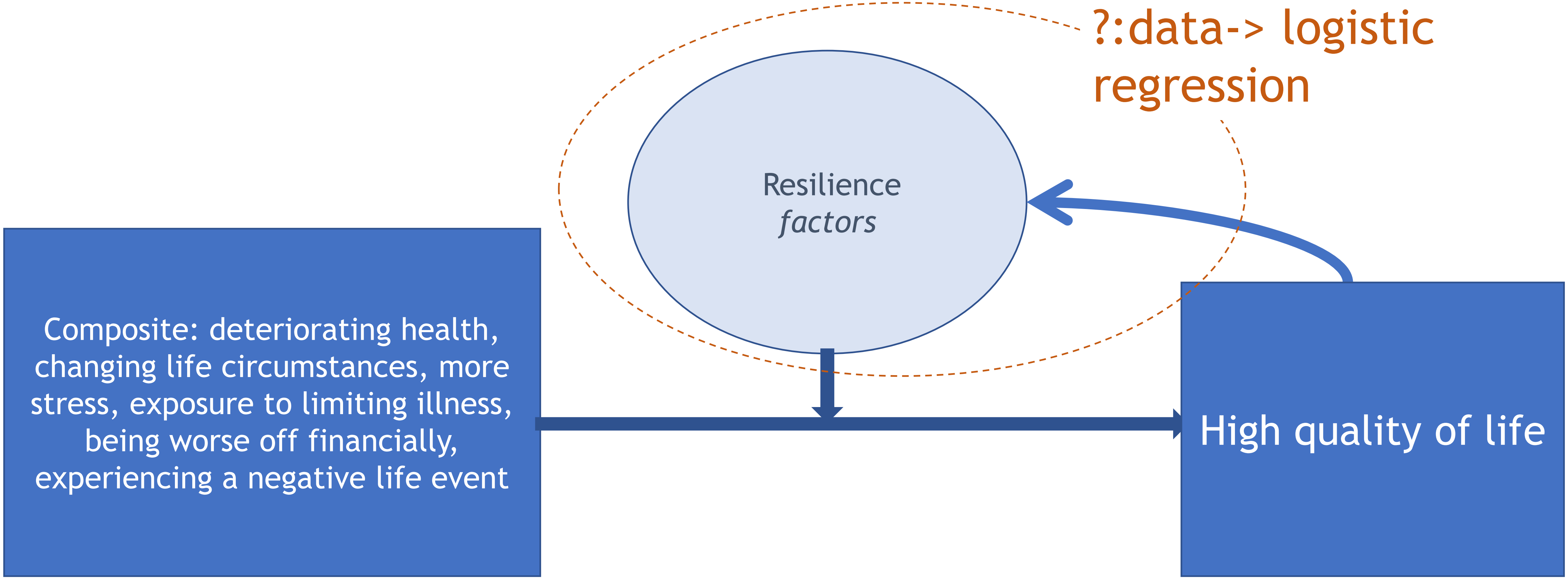

Example: qualitative *Kok et al. 2018*

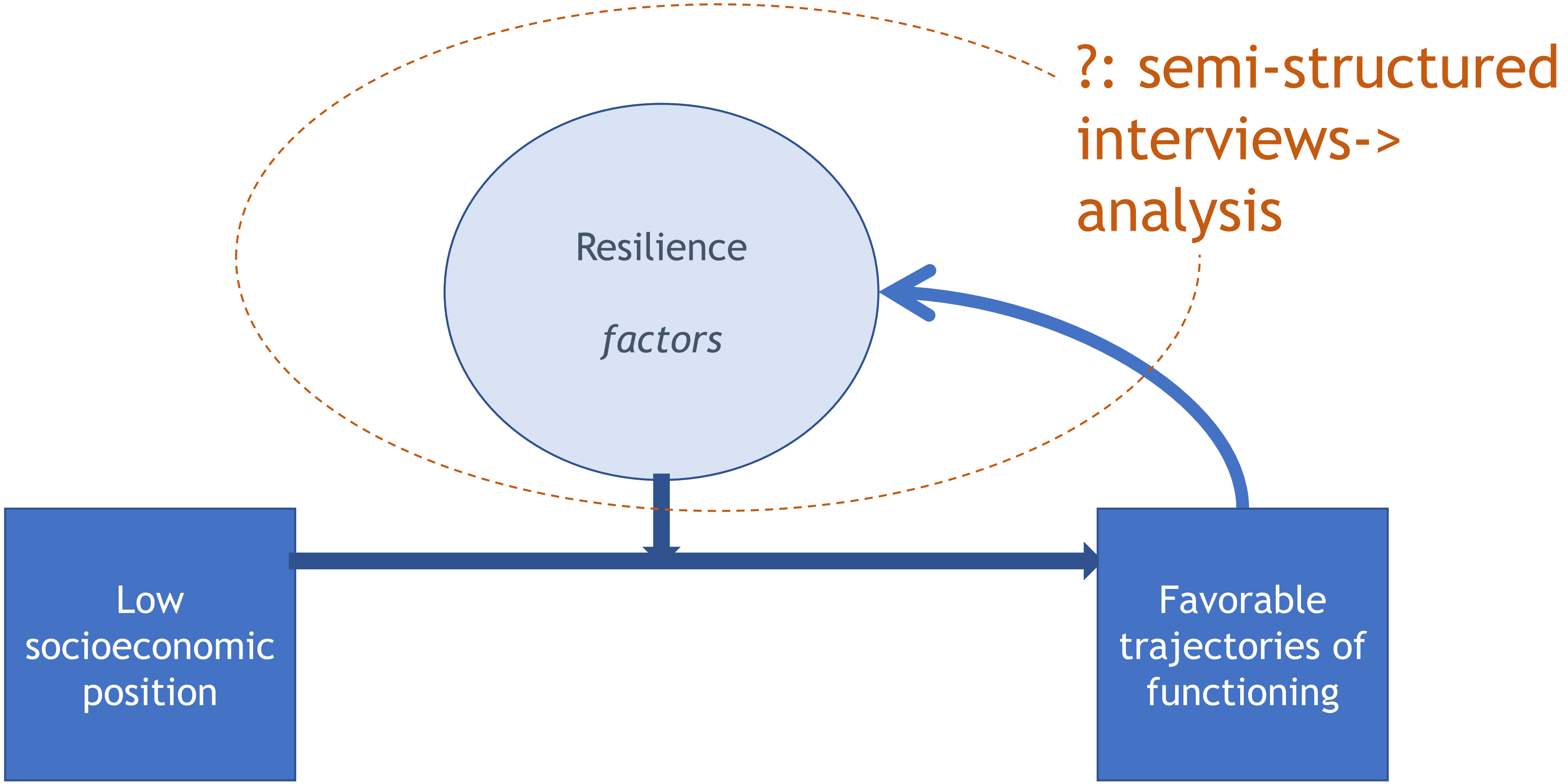

Factors

- Coping strategies
- Positive emotions/optimism
- Having a life goal/ caring for others/generativity
- Spirituality
- (Self-rated) health
- Physical activity
- Social support

Kessel 2013, Macleod 2016

Resilience

- Two approaches
    - Dynamical systems approach
        - Based on times series of functioning
            - Adversity not defined: naturally (constantly) occurring perturbations
            - Outcome: DIORs
    - A-priori
        - Based on definitions of:
            - Adversity
            - Good outcome
- Focus group: value approaches for research/ practice

References

- Gijzel SMW, van de Leemput IA, Scheffer M, Roppolo M, Olde Rikkert MGM, Melis RJF. Dynamical Resilience Indicators in Time Series of Self-Rated Health Correspond to Frailty Levels in Older Adults. *J Gerontol A Biol Sci Med Sci* (2017) 72(7):991-6.
- Gijzel SMW, van de Leemput IA, Scheffer M, van Bon GEA, Weerdesteyn V, Eijsvogels TMH, et al. Dynamical indicators of resilience in postural balance time series are related to successful aging in high-functioning older adults. *J Gerontol A Biol Sci Med Sci* (2018). Epub 2018/07/28. doi: 10.1093/gerona/gly170.
- Hildon, ZMA; Montgomery, SM; et al. Examining Resilience of Quality of Life in the Face of Health-Related and Psychosocial Adversity at Older Ages: What is “Right” About the Way we Age? The gerontologist. 2009: 50(1): 36-47.
- Infurna, FJ; Luthar, SS. The Multidemsional Nature of Resilience to Spousal Loss. J Pers Soc Psychol. 2017 June; 112(6):926-947.
- Van Kessel, G. The ability of older people to overcome adversity: A review of the resilience concept. Geriatric Nursing. 2013: 34(2):122-127.
- Kok, AAL; van Nes, F; Deeg, D.J.H.; Widdershoven, G; Huisman, M. “Tough Times Have Become Good Times”: Resilience in Older Adults With a Low Socioeconomic Position. Gerontologist, 2018, 58(5): 843-852.
- MacLeod S, Musich S , Hawkins K , et al. The impact of resilience among older adults. Geriatr Nurs 2016;37:266–72.
- Whitson HE, Duan-Porter W, Schmader KE, Morey MC, Cohen HJ, Colon-Emeric CS. Physical resilience in older adults: Systematic review and development of an emerging construct. *J Gerontol A Biol Sci Med Sci* 2016;71A:489–495.
